# New combinations and synonyms in discoid caespitose Andean *Senecio* (Senecioneae, Compositae)

**DOI:** 10.3897/phytokeys.132.38534

**Published:** 2019-10-03

**Authors:** Joel Calvo, Arturo Granda, Vicki A. Funk

**Affiliations:** 1 Instituto de Geografía, Facultad de Ciencias del Mar y Geografía, Pontificia Universidad Católica de Valparaíso, Avenida Brasil 2241, 2362807 Valparaíso, Chile Pontificia Universidad Católica de Valparaíso Valparaíso Chile; 2 Herbario del Departamento Académico de Biología, Facultad de Ciencias, Universidad Nacional Agraria La Molina, Av. La Molina s/n, apartado 12-056, Lima 12, Perú Universidad Nacional Agraria La Molina Lima Peru; 3 US National Herbarium, Department of Botany, Smithsonian Institution, Washington D.C., USA US National Herbarium, Department of Botany, Smithsonian Institution Washington United States of America

**Keywords:** Asteraceae, Bolivia, Chile, dichotomous key, Peru, taxonomy, *Werneria*

## Abstract

The names *Werneriamelanandra* and *W.pygmophylla* are transferred to the genus *Senecio*. They belong to the group of the discoid caespitose Andean *Senecio*, specifically to the subgroup with blackish anthers and style branches and whitish corollas. The recognition of *S.digitatus* as a distinct species is also discussed. Within the framework of the mentioned group, the names *S.casapaltensis* and *S.macrorrhizus* are lectotypified, S.humillimusvar.melanolepis is neotypified, an epitype is designated for the name *W.melanandra*, and nine new synonyms are proposed. An updated comprehensive dichotomous key including all discoid caespitose *Senecio* species from Bolivia and Peru is provided.

## Introduction

The discoid caespitose Andean *Senecio* L. species have traditionally been placed within S.subser.Caespitosi (O. Hoffm.) Cabrera & S.E. Freire (Freire et al. 2014). This infrageneric group was conceived for embracing the strictly caespitose species but also suffrutescent plants. As circumscribed by Cabrera et al. (1999), it includes ca. 50 species from southern South America. The infrageneric classification of *Senecio* at the subserial rank has been proposed for the Argentinian species, which are reasonably well-known (Freire et al. 2014). In Bolivia and Peru, by contrast, the understanding of the genus is poorer; the infrageneric classification remains barely resolved and several species remain misplaced. This is the case for two species that were hitherto recognized as members of *Werneria* Kunth, a genus morphologically similar to *Senecio* that can be differentiated by the combination of the following characters: involucral bracts fused at the base, absence of genuine supplementary bracts (calyculus), achene trichomes not myxogenic, rosettiform habit rather than caespitose. However, some *Senecio* species sometimes also have the involucral bracts partially fused at the base or do not have supplementary bracts. Such overlapping means that historically some species have been interchangeably treated as *Senecio* or *Werneria*, depending on the authors’ concepts. This is the case with *S.wernerioides* Wedd., a species that Grisebach (1874) and Kuntze (1898) transferred to *Werneria* but that is presently widely accepted as a heterotypic synonym of *S.breviscapus* DC. (Cabrera 1985; Beck and Ibáñez 2014; Freire et al. 2014). Similarly, S.repensvar.macbridei (Cuatrec.) Cabrera was initially described at the specific rank as *W.macbridei* Cuatrec. In Chile, Ricardi and Marticorena (1964) described *S.pfisteri* Ricardi & Martic., a species that has been recently synonymized with *Xenophyllumesquilachense* (Cuatrec.) V.A. Funk [≡*Werneriaesquilachensis* Cuatrec.] (Calvo et al. 2018). Such disparate treatments highlight the taxonomic complexity of these groups and indicate that some species are difficult to assign to one or another genus. In these cases, a detailed study based on the aforementioned set of characters is needed. In addition, the achene indumentum type appears to be a useful character for a proper identification. In arid regions *Senecio* species with myxogenic trichomes (with mucilaginous properties when soaked in water) are common (Nordenstam et al. 2009; Mukherjee and Nordenstam 2012). This character is also found in other genera within the tribe, e.g., *Dauresia* B. Nord. & Pelser, *Dolichoglottis* B. Nord., *Euryops* (Cass.) Cass. (Nordenstam et al. 2009), but it has not been reported in *Werneria*. Indeed, most species belonging to this genus have glabrous achenes or rarely scattered long trichomes near the base. Only *W.nubigena* Kunth usually displays achenes with dense, villous indumentum. It is composed of twin filiform trichomes, ca. 0.7 mm long, with acute to subacute, asymmetrical, usually forked apex, but does not exude mucilage when treated in water. On this basis, the myxogenic trichomes appear to be absent in *Werneria*, and therefore, it is another useful character to discriminate between the two genera.

Herein, we transfer *W.melanandra* Wedd. and *W.pygmophylla* S.F. Blake to the genus *Senecio*. Furthermore, and in disagreement with previous treatments (Rockhausen 1939; Cabrera 1949; Freire et al. 2014), we believe that *W.pygmophylla* and *S.digitatus* Phil. correspond to two different taxonomic entities and we justify this here accordingly. These species belong to a group of discoid caespitose Andean *Senecio* with blackish anthers and style branches and whitish corollas but differ from one another in some characters (see discussions below). Detailed illustrations and pictures are provided for each species, as well as a dichotomous key including the discoid caespitose *Senecio* species from Bolivia and Peru.

## Materials and methods

This contribution is the result of an intensive review of the published bibliography and the revision of herbarium specimens kept at BOLV, CONC, HSP, LPB, MA, MOL, SGO, US, and USM. Additionally, digital herbarium specimens from LP and P were studied; herbarium acronyms follow Thiers (2018). A light microscope was used for examination of microcharacters. Field work was conducted in Bolivia, southern Peru, and northern Chile.

## Results

### New combinations

#### 1. Senecio melanandrus

Taxon classificationPlantaeAsteralesAsteraceae

(Wedd.) J.Calvo, A.Granda & V.A.Funk comb. nov.

05FC566A-064F-5AAD-9E4D-8DD696C526C4

urn:lsid:ipni.org:names:60479386-2

**Figure 1.**
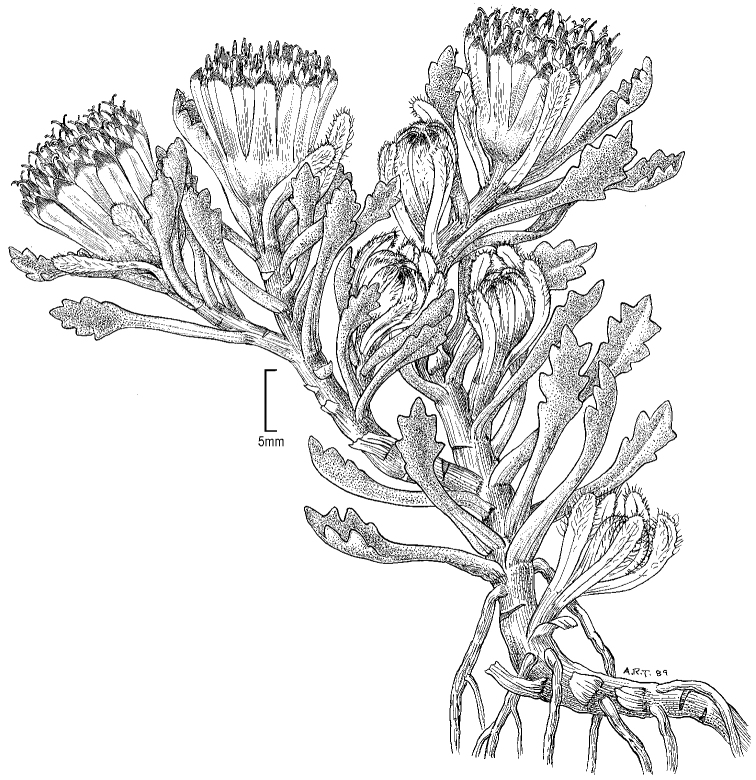
*Seneciomelanandrus*. Habit (drawn from *Buchtien 1589*). Illustration by Alice Tangerini.

**Figure 2.**
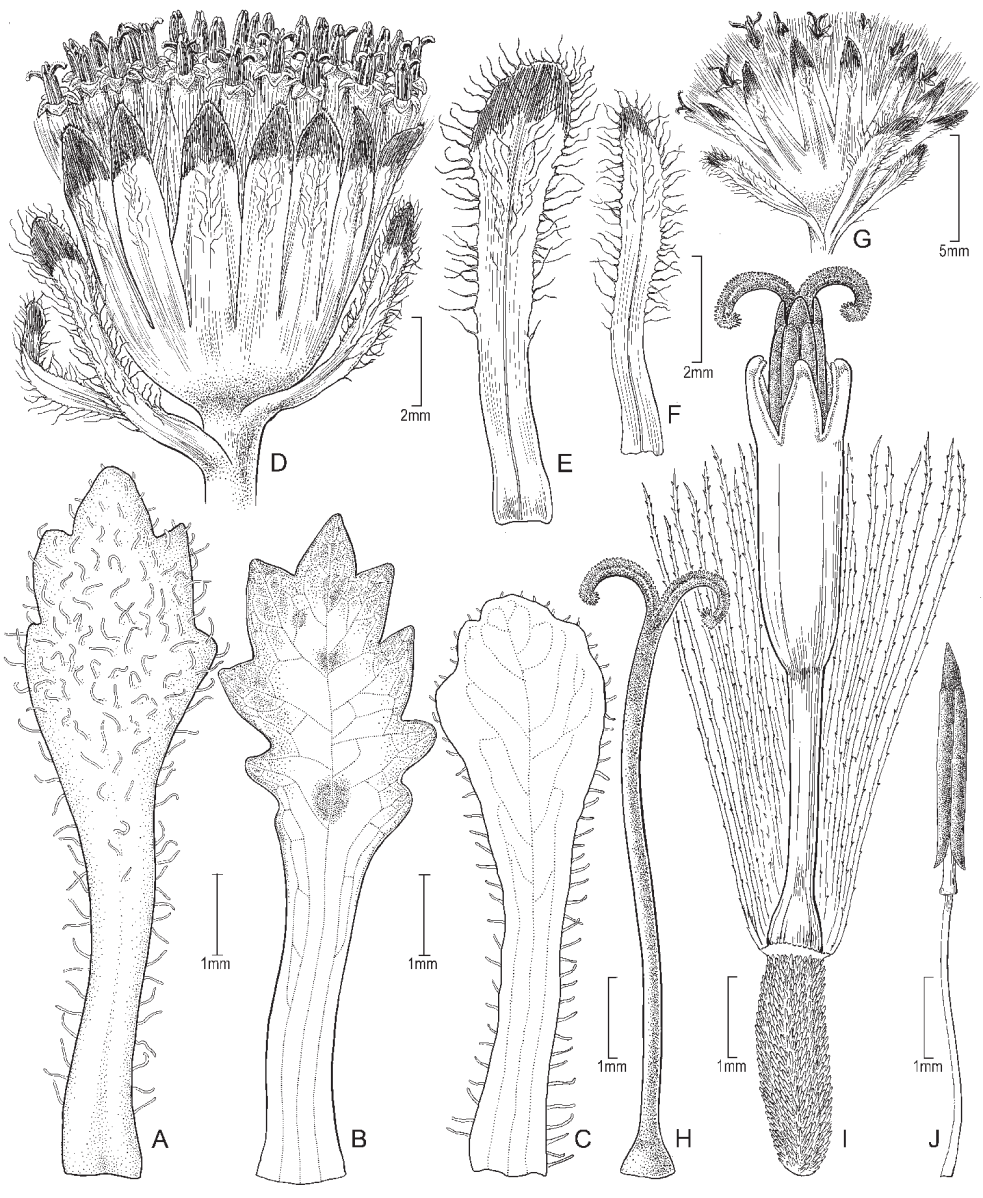
*Seneciomelanandrus***A–C** variability of leaves **D** capitulum **E, F** supplementary bracts **G** involucre **H** style **I** achene and floret **J** stamen. All details drawn from *Weberbauer 5446* except for **A** (drawn from *Calvo & Zárate 7872*), **C** (drawn from *Montesinos 3103*), and **G** (drawn from *Buchtien 1589*). Illustration by Alice Tangerini.

**Figure 3.**
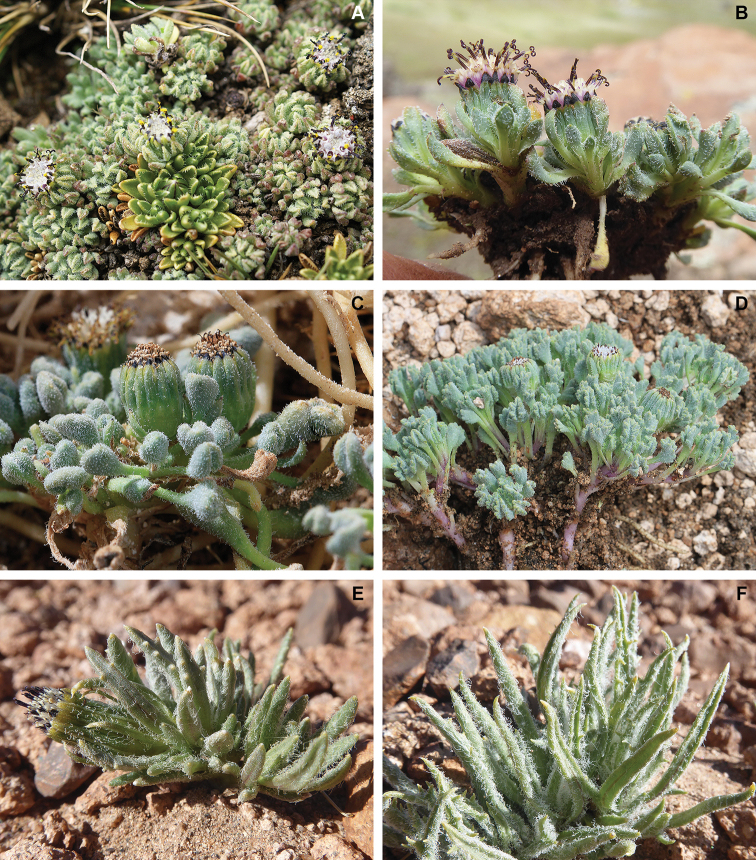
**A, B***Seneciomelanandrus* (pilose form) **C, D***Seneciopygmophyllus***E, F***Seneciodigitatus***A** habit (Peru, Cusco, Sibinacocha; *Meneses et al. 6968*) **B** leaves (Bolivia, Potosí, Kari Kari; *Calvo & Zárate 7872*) **C** habit (Chile, Tarapacá, Colchane; *Moreira-Muñoz 2876*) **D** leaves (Chile, Arica-Parinacota, Las Cuevas; *Moreira-Muñoz & Luebert 2470*) **E** habit **F** leaves (Chile, Antofagasta, Pacana; *Calvo 7926*). Picture **A** by Jim Farfán **B, E, F** by Joel Calvo **C, D** by Andrés Moreira-Muñoz.

**Figure 4.**
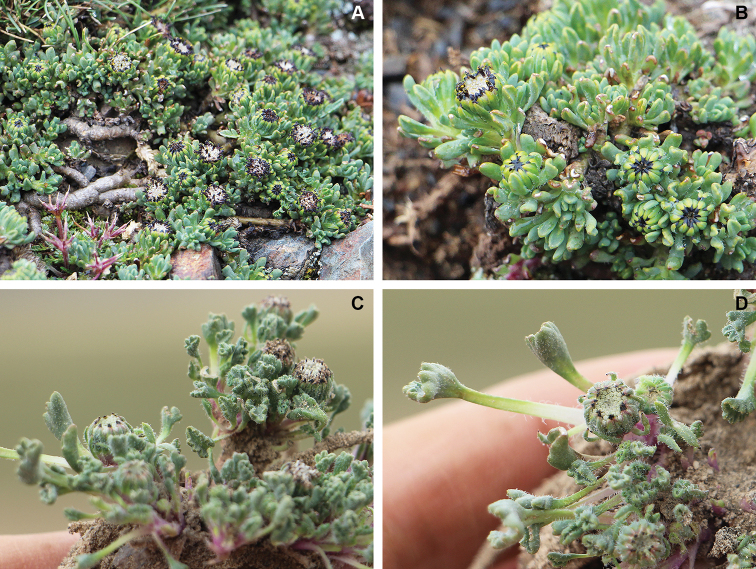
**A, B***Seneciomelanandrus* (glabrous form) **C, D***Seneciopygmophyllus***A** habit **B** leaves (Peru, Puno, pr. Ananea; *Funk et al. 13184*) **C** habit **D** leaves (Peru, Moquegua, pr. Anillune; *Funk et al. 13153*). Pictures by Mauricio Diazgranados.

Figs 1 , 2 , 3A, B , 4A, B

Werneria melanandra Wedd., Chlor. Andina 1: 88. 1856. Type: Bolivia. La Paz: ravin de Chuquiaguillo, 1851, H.A. Weddell s.n. (lectotype, designated by Rockhausen (1939) as “type”, pg. 284: P [P04319315]). Epitype, designated here: Bolivia. La Paz: am Chacaltaya (30 km von La Paz), 4800 m, Feb 1908, O. Buchtien 1589: US [US00622639]; isoepitype: US [US00622640].
Senecio humillimus var. melanolepis Wedd., Chlor. Andina 1: 104. 1856. Type: Bolivia. La Paz: Larecaja, viciniis Sorata, ad lacum Yuriguana, prope Anilaya, Ancumpampa, prope Ancohuma, 3800–5000 m, Apr 1860, G. Mandon 108 (neotype, designated here: GH [GH00012144]; isoneotypes: P [P03730752, P04370980], S [S-R-10871]), syn. nov.
Senecio vegetus var. lobatus Cabrera, Notas Mus. La Plata, Bot. 18(89): 222. 1955. Type: Bolivia. La Paz: Ingavi, Miriquiri, 4200 m, 10 Mar 1921, E. Asplund 2866 (holotype: S [not located, Arne Anderberg in litt.]), syn. nov.
Senecio pucapampaensis H. Beltrán, Arnaldoa 15: 212. 2009. Type: Peru. Huancavelica: Pucapampa, debajo de Chonta, 4500–4600 m, 9 May 1958, O. Tovar 2959 (holotype: USM-00277274), syn. nov.
Senecio sykorae Montesinos, PhytoKeys 39: 6. 2014. Type: Peru. Moquegua: General Sánchez Cerro, Yunga, E of Yunga, on the peaks of Perusa mountain, 16°11'08"S, 70°38'14"W, 4802 m, 13 Apr 2012, D. Montesinos & F. Calisaya 3805 (holotype: USM s.n.; isotype: HUSA n.v.), syn. nov.
Senecio tassaensis Montesinos, PhytoKeys 39: 11. 2014. Type: Peru. Moquegua: General Sánchez Cerro, Ubinas, cumbre nevada del cerro Pirhuani Querala, 4650 m, 16°09'S, 70°43'W, 7 Apr 2011, D. Montesinos 3103 (holotype: HUSA n.v.; isotypes: MOL n.v. [not located, likely never sent], USM-247549), syn. nov.
Senecio canoi P. Gonzáles, Montesinos & Ed. Navarro, Anales Jard. Bot. Madrid 72(2): 1. 2015. Type: Peru. Puno: Carabaya, Corani, Minaspata, arriba de Chacaconiza, 14°01'57"S, 70°41'54"W, 4999 m, 14 Apr 2014, P. Gonzáles 2989 (holotype: USM n.v.), syn. nov.
Senecio vegetus sensu Cabrera (1955, 1985), non Weddell (1856).

##### Description.

Caespitose perennial herb. Leaves 4–15 mm long, 1.2–2.6 mm wide, linear-oblong to spatulate, apex acute to obtuse, base narrowed, margins entire, crenate or dentate, conduplicate downwards (rarely flat), glabrous, with marginal trichomes on the narrowed base or densely pilose, somewhat fleshy, greenish or glaucous. Capitulum discoid, solitary, terminal, sessile or subsessile; involucre 5–8 mm long, 3.7–9 mm wide. Involucral bracts 11–16, oblong-lanceolate, 3.8–4.9 mm long, 0.9–1.8 mm wide, partially fused at the base, smooth, glabrous or with trichomes on the abaxial surface ca. 0.7 mm long, dark purple- or blackish-tipped. Supplementary bracts (calyculus) 2–4(–6), linear to slightly spatulate, 4.2–7.5 mm long, 0.5–1 mm wide, smooth, three-quarters to as long as the involucral bracts, with trichomes (rarely glabrous), dark purple- or blackish-tipped. Disc florets 20–45, 4.3–6.3 mm long, 0.8–1.1 mm wide, 5-lobed, conspicuously differentiated in a distinct tube and campanulate limb, whitish. Anther bases auriculate, clearly acute, dark purple to blackish; filament collar balusterform. Style branches truncate with a crown of sweeping hairs, dark purple to blackish. Achenes 2.1–2.2 mm long, ca. 0.5 mm wide, brownish, covered by dense indumentum of obtuse whitish myxogenic twin trichomes ca. 0.2 mm long; pappus 5–6 mm long, barbellate, whitish. Chromosome number: unknown.

##### Additional iconography.

Beltrán (2008: pg. 216, fig. 2, sub *S.pucapampaensis*); Montesinos-Tubée (2014: pg. 7, fig. 2; pg. 13, fig. 4B, sub *S.sykorae*); Montesinos-Tubée (2014: pg. 12, fig. 3; pg. 13, fig. 4C, sub *S.tassaensis*); Montesinos-Tubée et al. (2015: pg. 2, fig. 1; pg. 3, fig. 2, sub *S.canoi*).

##### Distribution and habitat.

**Figure 5.**
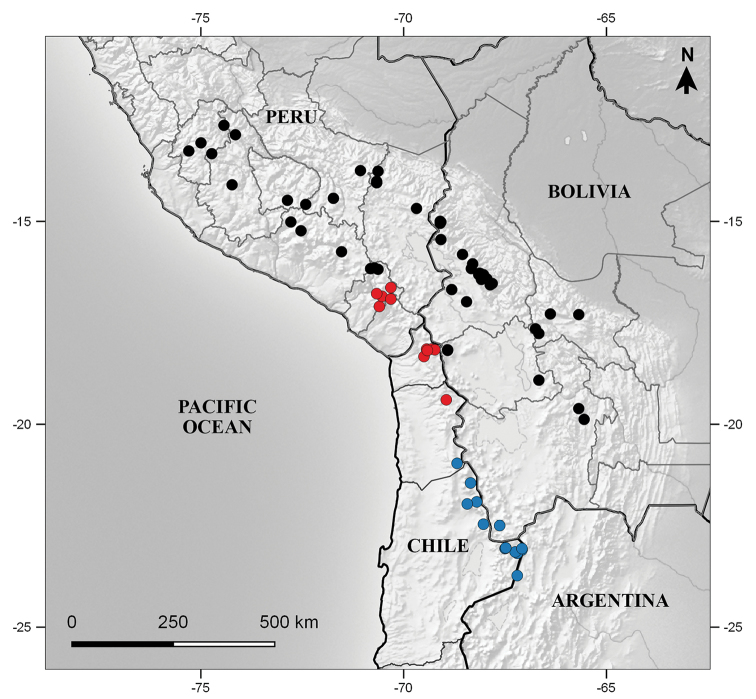
Distribution map of *Seneciomelanandrus* (black circle), *S.pygmophyllus* (red circle), and *S.digitatus* (blue circle).

Bolivia (Cochabamba, La Paz, Oruro, Potosí) and Peru (Apurímac, Arequipa, Ayacucho, Cusco, Huancavelica, Moquegua, Puno) (Fig. 5). It grows in exposed places mainly in the subhumid and dry puna ecoregions, at elevations of 3800–5100 m.

##### Phenology.

Flowering mainly from January to June, although some flowering specimens have been collected in November.

##### Etymology.

The epithet *melanandrus* means having dark or black stamens, which describes a striking character of this species.

##### Discussion.

This species is transferred to *Senecio* on the basis that it has genuine supplementary bracts (calyculus), the involucral bracts are not clearly fused at the base, it displays a caespitose habit with short stems, and it has myxogenic achene trichomes. Furthermore, its morphologically most similar species are currently treated as *Senecio* members: i.e., *S.digitatus*, *S.madidiensis* J. Calvo & A. Fuentes, *S.pygmophyllus* (see new combination below), and *S.scorzonerifolius* Meyen & Walp. All the names included in the synonymy were also described within the genus *Senecio*.

*Seneciomelanandrus* is a highly variable species that has been variously interpreted. The poor condition of the type material probably helped to maintain the uncertainty surrounding the application of this name. Weddell (1856) described the leaves as “integerrimis vel nonnullis dente triangulari, […] glabriusculis vel inconspicue ciliolatis” [entire or with a few triangular teeth, rather glabrous or inconspicuously ciliate]. Several years later Rockhausen (1939), who published the first comprehensive taxonomic revision of the genus *Werneria*, stated that the leaves have “marginibus obsolete glanduloso-ciliolata” [margins scarcely glandular-ciliate]. On the basis of our studies, this species displays an unusually wide variability in leaf margin and indumentum of leaves and involucre, which is reflected in the number of names included in the synonymy. The leaf margin may be entire, crenate or dentate, variability that can be even found in the same individual. Likewise, the leaf indumentum varies from densely pilose (Fig. 3A, B) to almost glabrous (Fig. 4A, B). In Bolivia, the pilose forms are common although some glabrescent specimens are found near Nevado Sajama (*Liberman 821*, LPB, US) and in northern La Paz Department (*Menhofer 1901*, US). The glabrous forms also appear in the Peruvian regions of Huancavelica, northern Ayacucho, and northern Puno, and were recently treated under the names *S.pucapampaensis* H. Beltrán and *S.canoi* P. Gonzáles & al. [see Calvo and Fuentes (2018)]. These glabrous plants usually have dentate leaves, but forms exist with rather subentire leaves (*Gonzáles 3568*, USM). The dentate, pilose forms that are frequently found in Bolivia were described as *S.tassaensis* Montesinos on the basis of a single collection from Moquegua (southern Peru). From the same region, a form with almost entire, glabrous leaves was named *S.sykorae* Montesinos. This form was also collected near the Bolivian locality of Ulla Ulla (*Menhofer 1901*, US). This puzzling distribution pattern and a continuum of intermediates suggest that these forms do not deserve taxonomic recognition. Despite this variability, *S.melanandrus* is well characterized by supplementary bracts that are almost as long as the involucral bracts, the leaf lamina narrowed at the base, the blackish anthers and style branches, the whitish corollas, and by its myxogenic achene trichomes. The apex of the involucral bracts is usually remarkably dark-colored. Indeed, the epithet *melanolepis* of Weddell’s varietal name, here included in the synonymy, explicitly refers to this character, i.e., having black scales (involucral bracts). It is noteworthy that the anther bases were hitherto described as obtuse; however, they are auriculate and clearly acute.

The name Seneciovegetusvar.lobatus Cabrera, here synonymized with *S.melanandrus*, was included by Cabrera (1985) in the synonymy of *S.vegetus* (Wedd.) Cabrera. Cabrera (1955, 1985) described this latter species as having silky-pubescent achenes. We had the opportunity of studying some of the specimens that he examined and they indeed correspond to *S.melanandrus* (i.e., *Beck 7952*, *Mandon 108*, *Menhofer 2013*, *Weberbauer 7491*). Cabrera’s interpretation of *S.vegetus* (≡S.humillimusvar.vegetus Weddell) might be explained by the fact that one of the syntypes (P [P01816588]) contains mixed material and some plants certainly correspond to *S.melanandrus* (the individual on the left hand and likely the fragment at the right hand below). The syntype P [P01816917], not containing admixtures, shows plainly glabrous plants with the leaves entire and obtuse at the apex. Therefore, and in disagreement with Cabrera, we consider that *S.vegetus* and *S.melanandrus* correspond to distinct taxonomic entities. The former belongs to the subgroup with yellowish anthers, style branches, and corollas whereas the latter is a member of the subgroup displaying blackish anthers and style branches and whitish corollas. However, it is important to point out that the accurate taxonomic position of *S.vegetus* remains uncertain. Because of the number of involucral bracts, the leaf morphology, the yellowish corollas, and the presumed glabrous achenes, we believe that this taxon is related to *S.gamolepis* Cabrera. Additional studies are needed to establish its correct taxonomic position. For the time being, we prefer not including it in the key.

Our efforts to locate the type material of S.humillimusvar.melanolepis Wedd. were unsuccessful. In fact, all the collections cited in the protologue that were located correspond to the other varieties described by Weddell. For that reason, we selected as the neotype a Mandon collection that perfectly matches the diagnosis provided by Weddell. Moreover, it was identified as S.humillimusvar.melanolepis by Schultz Bipontinus [see Mandon (1865)], which supports our interpretation of this taxon. The specimen P03730757 is excluded because it contains mixed material.

The holotype of the name *S.canoi* should be housed at USM (Montesinos-Tubée et al. 2015); however, it was not located. The paratype *Gonzáles 3429* (USM) was also not located at USM. As a result, we studied the collections *Gonzáles 3428* and *Gonzáles 3441* (USM), both collected around the *locus classicus* on the same day as the paratype. Likewise, the holotype of Seneciovegetusvar.lobatus appears to be missing (Arne Anderberg in litt.). Cabrera indicated as paratype the collection *Mandon 108*, which is here selected as neotype for S.humillimusvar.melanolepis.

Finally, in order to remove any uncertainty surrounding the application of this name, and considering that the conditions of the type material are deficient for a proper study of the diagnostic characters, we consider it appropriate to designate an epitype. The selected specimen is *Buchtien 1589* (US00622639) from Chacaltaya, a mountain not far from the *locus classicus* of *W.melanandra*. A duplicate was found at US.

##### Selected specimens examined.

**BOLIVIA. Cochabamba**: Arque, Cruce Ventilla, 17°46'S, 66°40'W, 17 May 1981, O. Murgia 276 (LPB); cordillera del Tunari, cumbres del cerro Tunari, 17°17'S, 66°23'W, 25 Mar 1990, G. Navarro 653 (BOLV); Tapacarí, arriba rancho Wacakhariña, 3 km al NE de Japo K’asa (km 125 Cbba-Oruro), 17°39'S, 66°45'W, 9 Mar 1995, H.U. Pestalozzi 446 (BOLV); Tiraque, P.N. Carrasco, cordillera Juno, 17°18'S, 65°41'W, 18 Mar 2001, M. Zárate & D. Méndez 1087 (LPB); **La Paz**: Murillo, La Paz 32 km hacia Unduavi, 16°19'S, 68°2'W, 3 Apr 1983, S.G. Beck 7952 (LPB); Murillo, camino La Paz-Lambate, cerca Apacheta entrando al desvío hacia el Illimani, 2 km entrando hacia Milla Milla, 16°34'S, 67°52'W, 6 Apr 2012, S.G. Beck 32782 (LPB); Murillo, La Paz subiendo el valle Kaluyo hasta el albergue ecoturístico Pampalarama, 16°19'S, 68°4'W, 22 Mar 2009, S.G. Beck 33091 (LPB); Murillo, subiendo el valle de Irpavi hasta Palcoma, subiendo el río Hati Jahuira, 16°25'S, 67°57'W, 26 Apr 2013, S.G. Beck 34141 (LPB); Los Andes, above cumbre (pass) on rd. through Hichu-Kkota valley on rd. to mina La Fabulosa, 21 km from base of lag. Khara Kkota, 16°10'S, 68°20'W, 29 Apr 1995, V.A. Funk 11406 (US; the duplicate at LPB corresponds to *Werneriaapiculata* Sch. Bip.); Murillo, Zonga valley, laguna Pata Kkota, 1.5 km S of pass, 16°18'S, 68°7'W, 11 Apr 1995, V.A. Funk & N. Bernal 11270 (LPB, US); Murillo, nev. Huayna Potosí, E slopes above rd., 16°17'S, 68°8'W, 12 Apr 1995, V.A. Funk & N. Bernal 11284A (US); Franz Tamayo, Canhuma (Ulla-Ulla), subiendo al cerro Laramani, 15°0'S, 69°6'W, 22 Jan 1983, X. Menhofer 1901 (US); Franz Tamayo, estancia Okaria (Ulla-Ulla), 15°3'S, 69°6'W, 24 Feb 1983, X. Menhofer 2013 (LPB); Murillo, 3.4 km N of Milluni on road to Zongo, 16°18'S, 68°7'W, 25 Apr 1985, J.C. Solomon & M. Moraes 13440 (LPB, US); Ingavi, cantón Jesús de Machaca, comunidad Titicani-Tacaca, a 20 km de Guaqui, 16°41'S, 68°49'W, 8 Apr 1989, X. Villavicencio 457 (LPB); **Oruro**: Eduardo Abaroa, Challapata, comunidad Churacani, cerca a la laguna Chullumpiri, 18°55'S, 66°40'W, 1 Apr 2018, M. Guzmán 125 (LPB); Sajama, nevado de Sajama, sur del cerro Jasasuni [Asa-asuni], 18°11'S, 68°55'W, 18 Mar 1984, M. Liberman 821 (LPB, US); Sajama, cantón Sajama, 18°10'S, 68°55'W, 17 Feb 1998, F. Loza de la Cruz 315 (LPB); **Potosí**: cordillera Kari Kari, aprox. 3.2 km arriba de la laguna San Sebastián, 19°37'S, 65°41'W, 13 Feb 2019, J. Calvo & M. Zárate 7872 (BOLV); José M. Linares Lizarazu, comunidad Alkatuyo, cerro Ichurata, 53 km SE de Potosí, 14 km al N de la escuela de Alkatuyo, 19°53'S, 65°33'W, 22 Jan 1994, F. Marino 309 (LPB). **PERU. Apurímac**: Antabamba, Juan Espinoza Medrano, paraje Ccanccahuane a 18 km al S de la comunidad campesina de Mollebamba, zona Minaminayoc, 14°29'S, 72°52'W, 5 Jun 2017, B. Espinoza-Prieto 534 (USM); **Arequipa**: pr. Chivay, ladera S del nevado Huarancante, 15°45'S, 71°32'W, 1 Apr 2005, C. Aedo & A. Galán 11022 (MA, USM); Castilla, Orcopampa, minas de Poracota, cerca a quebrada Faculla, 15°14'S, 72°32'W, 20 Apr 2011, H. Beltrán 7112 (USM); La Unión, Huaynacotas, Sarajorepampa, 15°1'S, 72°47'W, 18 Mar 2011, D. Montesinos 2949 (USM); **Ayacucho**: Huanca Sancos, Putajasa, 14°6'S, 74°14'W, 24 Feb 2002, A. Cano et al. 11963 (USM); Huanta, mt. Razuhuilca, 12°52'S, 74°9'W, 4–6 Feb 1926, A. Weberbauer 7491 (CONC, F); **Cusco**: Chumbivilcas, Santo Tomás, compañía minera Azuca (borde departamental Cusco-Apurímac), 14°35'S, 72°25'W, 13 Apr 2011, H. Beltrán 7032 (USM); Velille, Uchucarco, cerca a Soracocha, 14°26'S, 71°44'W, 23 Apr 2015, P. Gonzáles 3600 (USM); Velille, Uchucarco, cerca a Soracocha, 14°26'S, 71°44'W, 23 Apr 2015, P. Gonzáles 3601 (USM); cordillera de Vilcanota, cuenca de la laguna Sibinacocha, cerro Rititica, 13°45'S, 71°4'W, 5 Mar 2019, R.I. Meneses et al. 6968 (LPB); **Huancavelica**: Huaytará, Pilpichaca (abra Apacheta), 13°20'S, 74°44'W, 4 Jul 2010, A. Cano, W. Mendoza & A. Delgado 19680 (USM); Huachocolpa, alrededores de la unidad minera Caudalosa, 13°4'S, 75°0'W, 23–31 Mar 2015, P. Gonzáles 3568 (USM); Castrovirreyna, cordillera between Pisco and Ayacucho, 13°16'S, 75°18'W, May 1910, A. Weberbauer 5446 (F, GH); **Moquegua**: General Sánchez Cerro, Ubinas, S of Pillone, 16°10'S, 70°49'W, 24 Mar 2013, D. Montesinos 4023 (USM); General Sánchez Cerro, Ubinas, Matazo, 16°10'S, 70°49'W, 28 Mar 2015, D. Montesinos 4242 (USM); General Sánchez Cerro, Ubinas, Querala, 16°10'S, 70°49'W, 2 Mar 2018, D. Montesinos 5918 (USM); **Puno**: just W of abra on unpaved track, ca. 17 km from Puno-Ananea rd., 14°41'S, 69°41'W, 16 Mar 2014, V.A. Funk, M. Diazgranados & E. Cochachin 13184 (US, USM); Carabaya, Corani, Chacaconiza, 14°1'S, 70°40'W, 14 Jan 2015, P. Gonzáles 3428 (USM); Carabaya, Corani, Chacaconiza, 14°3'S, 70°40'W, 14 Jan 2015, P. Gonzáles 3441 (USM); Carabaya, Corani, Chacaconiza, 14°3'S, 70°40'W, 14 Jan 2015, P. Gonzáles 3444 (USM); Carabaya, alrededores de Condena, 13°46'S, 70°38'W, 9 Nov 2017, H. Trinidad 4192 (USM).

#### 2. Senecio pygmophyllus

Taxon classificationPlantaeAsteralesAsteraceae

(S.F. Blake) J.Calvo, A.Granda & V.A.Funk comb. nov.

0E069DFA-4C68-51DB-AAB2-94C157EB9832

urn:lsid:ipni.org:names:60479387-2

**Figure 6.**
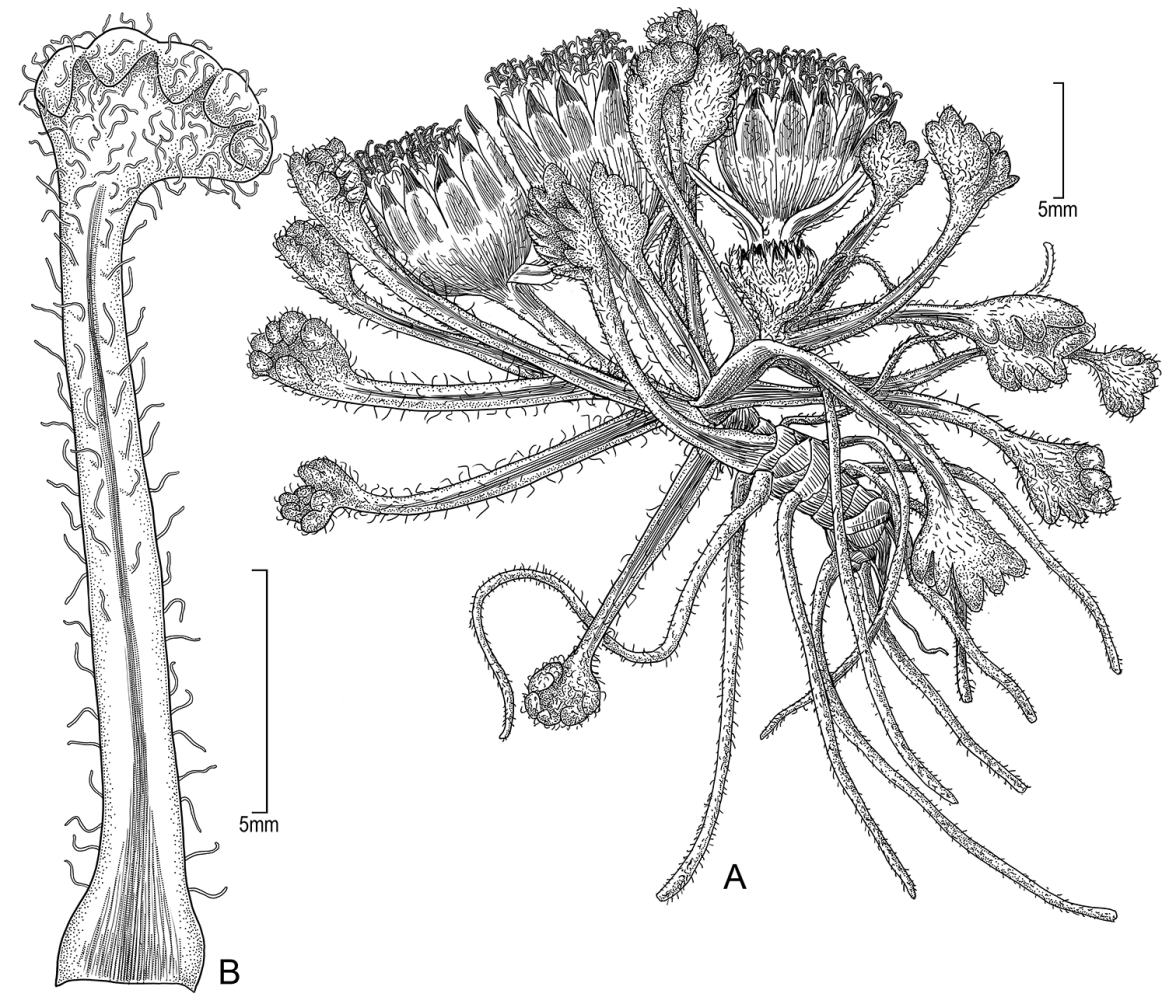
*Seneciopygmophyllus***A** habit (drawn from *Funk et al. 13153*) **B** leaf (drawn from *Weberbauer 7358*). Illustration by Alice Tangerini.

Figs 3C, D , 4C, D , 6

Werneria pygmophylla S.F. Blake, J. Washington Acad. Sci. 18: 491. 1928. Type: Peru. Moquegua: cordillera East of Carumas, 4500–4600 m, 7–8 Mar 1925, A. Weberbauer 7358 (holotype: F [F-552587]; isotypes: CONC [CONC-28864], G [G00356025], US [US00622822]).
Senecio laucanus Ricardi & Martic., Gayana, Bot. 11: 17. 1964. Type: Chile. Arica-Parinacota: camino de Putre a Chucuyo, km 17, 4250 m, 12 Feb 1964, C. Marticorena, O. Matthei & M. Quezada 208 (holotype: CONC [CONC-29864]; isotype: CONC), syn. nov.

##### Description.

Caespitose perennial herb. Leaves long pseudopetiolate; leaf lamina 2.5–5.5 mm long, 2.4–5.5 mm wide, ovate to suborbiculate, obtuse at the apex, rounded to truncate at the base, typically crenate-lobate with 3–9 rounded lobes, revolute, usually strongly conduplicate downwards, pilose on both surfaces, somewhat fleshy, glaucous; pseudopetiole 5–25 mm long, flat, slightly broadened at the base, marginally ciliate. Capitulum discoid, solitary, terminal, sessile or subsessile; involucre 6–8 mm long, 7–10 mm wide. Involucral bracts 16–21, oblong-lanceolate, 2.5–4 mm long, 0.7–1.7 mm wide, partially fused at the base, smooth, with trichomes on the abaxial surface 0.5–0.8 mm long, dark purple- or blackish-tipped. Supplementary bracts ca. 3, linear, 6–7.5 mm long, 0.5–0.8 mm wide, smooth, three-quarters to as long as the involucral bracts, with trichomes on the margins, dark purple- or blackish-tipped. Disc florets 50–82, 3.5–5.1 mm long, 0.6–1 mm wide, 5-lobed, conspicuously differentiated in a distinct tube and campanulate limb, whitish. Anther bases auriculate, clearly acute, dark purple to blackish; filament collar balusterform. Style branches truncate with a crown of sweeping hairs, dark purple to blackish. Achenes 1.7–1.8(–2.5) mm long, ca. 0.5 mm wide, brownish, covered by dense indumentum of obtuse whitish myxogenic twin trichomes ca. 0.2 mm long; pappus 3–4.5 mm long, barbellate, whitish. Chromosome number: unknown.

##### Additional iconography.

Blake (1928: pg. 496, fig. 1F, G, sub *W.pygmophylla*); Ricardi and Marticorena (1964: pg. 19, fig. 6, sub *S.laucanus*).

##### Distribution and habitat.

Chile (Arica-Parinacota, N Tarapacá) and Peru (Moquegua) (Fig. 5). The species is also expected in the Peruvian department of Tacna and in the Bolivian region bordering northern Chile, although no collections have been studied from there. It grows in exposed places on sandy soils, between elevations of 4100–4700 m.

##### Phenology.

Collected in bloom from January to June, although full bloom probably takes place between March and April.

##### Etymology.

The epithet refers to the resemblance of the leaves to a fist.

##### Discussion.

Blake (1928) placed his new species within *Werneria* arguing that the involucral bracts were connate half way. Otherwise, he assumed a close similarity between it and a *Senecio* species collected by Pennell here identified as *S.moqueguensis* Montesinos (see protologue of *W.pygmophylla*). It is certain that the involucral bracts of *S.pygmophyllus* are usually partially fused at the base; however, this character alone cannot be used as diagnostic to place a species in one or another genus. Rather, we prefer to base a decision on a set of characters, i.e., presence or absence of genuine supplementary bracts, involucral bracts free or fused at the base, achene trichomes myxogenic or not, and rosettiform or caespitose habit. Accordingly, we consider that this species should be placed within *Senecio* on the basis of the following characters: presence of supplementary bracts, myxogenic achene trichomes, and caespitose habit. This decision is also supported by the fact that it was inaccurately considered a synonym of *S.digitatus* for a long time.

Rockhausen (1939) was the first author who treated *W.pygmophylla* as a heterotypic synonym of *S.digitatus*. Since then, most authors followed his treatment (e.g., Cabrera 1949; Freire et al. 2014). Ricardi and Marticorena (1966), in disagreement, concluded that they correspond to two distinct taxonomic entities. We agree with Ricardi and Marticorena’s treatment after studying the respective type materials, further collections from southern Peru and northern Chile, and living plants. The two species can be differentiated by their leaf shape and indumentum type. *Seneciopygmophyllus* has a lamina clearly differentiated from the pseudopetiole (petioliform base); usually the lamina is remarkably reduced when compared with the pseudopetiole length (at least in the more basal leaves). The lamina we observed were ovate to suborbicular, typically crenate-lobate with 3–9 rounded lobes and revolute margins (Fig. 4C). In contrast, *S.digitatus*has linear to slightly spatulate leaves narrowed at the base (Fig. 3E, F). This latter species is extremely variable with regard to the leaf margin, which can be dentate, pinnatipartite or distantly pinnatisect, with clearly acute teeth; however, specimens with entire leaves and even individuals displaying both entire and dentate leaves were occasionally observed. The leaf apex is acute and usually shows a whitish callus-like tip, whereas in *S.pygmophyllus* the apex is always plainly obtuse and unadorned (Fig. 3C, 4D). Both species usually have abundant indumentum on the leaves, involucre, and supplementary bracts but the type of trichomes differs and is useful to separate them from one other. The indumentum of *S.pygmophyllus* is pilose whereas in *S.digitatus* the trichomes are clearly arachnoid, longer, and intermingled. Moreover, the indumentum of *S.digitatus* is essentially concentrated on the adaxial surface, whereas in *S.pygmophyllus* the leaf lamina has trichomes on both surfaces. Their distribution areas do not overlap (Fig. 5).

*Seneciopygmophyllus* might be confused with those forms of *S.melanandrus* displaying pilose, dentate leaves. A useful character to discriminate them from each other is the leaf shape, although some overlap has been detected in a few specimens. In *S.pygmophyllus* the leaves are clearly pseudopetiolate and the ratio lamina/pseudopetiole length usually is very low in the more basal leaves (Fig. 4D). In contrast, *S.melanandrus* displays a lamina progressively narrowed at the base (Fig. 3B). The distinctive pseudopetiole length of *S.pygmophyllus* might be an adaptation to the sandy soils where this species thrives because the plants usually appear to be partially sunken. Additionally, the number of disc florets tends to be higher in *S.pygmophyllus* (50–82 vs. 20–45), as well as the number of involucral bracts (16–21 vs. 11–16). Since the mentioned morphology coincides with geographical separation, we consider it appropriate to recognize it as a distinct species.

The name *S.laucanus* Ricardi & Martic. was described from northern Chile (Arica-Parinacota) and it was hitherto considered endemic to this country (Moreira-Muñoz et al. 2016). It is included in the synonymy of *S.pygmophyllus* since we failed to identify any diagnostic character to differentiate them. In some specimens from Chile the more basal leaves are not so long pseudopetiolate as in the typical forms (e.g., *Moreira-Muñoz & Luebert 2470*), but it is considered as part of the variability encompassed by this species; indeed, this morphology probably responds to the fact that these plants grow on less sandy soils.

##### Specimens examined.

***Seneciodigitatus*. ARGENTINA. Salta**: Los Andes, Huaitiquina, 23°44'S, 67°12'W, 27 Feb 1972, Cabrera et al. 22559 (LP). **BOLIVIA. Potosí**: Sud Lípez, a 1 km al W de salar Chalviri, 22°30'S, 67°38'W, 7 May 1999, N. Massi & C. Salles 726 (LPB) [first record for Bolivia]. **CHILE. Antofagasta**: El Loa, camino entre Ascotán y San Pedro de Conchi, 21°58'S, 68°26'W, 4 Apr 1985, M. Arroyo 85-606 (CONC); El Loa, cerro Losloyo, ladera SE, 23°9'S, 67°15'W, 9 Apr 1997, M. Arroyo, L. Cavieres & A. Humaña 97331 (CONC); El Loa, cerro Nevados de Poquis, ladera SO, 23°4'S, 67°5'W, 9 Apr 1997, M. Arroyo, L. Cavieres & A. Humaña 97343 (CONC); El Loa, pampa Laguna Helada, 23°6'S, 67°5'W, 9 Apr 1997, M. Arroyo, L. Cavieres & A. Humaña 97403 (CONC); El Loa, pampa Loyoques, 23°11'S, 67°12'W, 9 Apr 1997, M. Arroyo, L. Cavieres & A. Humaña 97408 (CONC); El Loa, cordón cerro de la Pacana, cuesta entre salar de Aguas Calientes y quebrada Quepiaco, 23°3'S, 67°29'W, 11 Apr 1997, M. Arroyo, L. Cavieres & A. Humaña 97477 (CONC); El Loa, cordón cerro de la Pacana, cuesta entre salar de Aguas Calientes y quebrada Quepiaco, 23°4'S, 67°30'W, 11 Apr 1997, M. Arroyo, L. Cavieres & A. Humaña 97498 (CONC); El Loa, Toconao, camino a Tara, monjes de La Pacana, 23°3'S, 67°29'W, 6 Mar 2019, J. Calvo 7926 (SGO); cruce camino internacional Paso Jama con camino a salar de Tara, 23°3'S, 67°29'W, 19 Dec 1996, A. Moreira-Muñoz 317 (SGO); Machuca-Copacoya, 22°28'S, 68°2'W, 18 Feb 1885, F. Philippi s.n. (LP, SGO); laguna de Llaillai, 21°55'S, 68°12'W, 23 Feb 1885, F. Philippi s.n. (CONC, LP, SGO, SI); El Loa, Ascotán, 21°27'S, 68°21'W, 23 Jan 1943, E. Pisano & J. Venturelli 1753 (SGO); El Loa, entre Machuca y Tatio, 15 Feb 1943, E. Pisano & J. Venturelli 1866 (CONC, SGO); **Tarapacá**: [without locality], Feb 1885, F. Philippi s.n. (K); Iquique, Collaguasi, San Carlos, 20°58'S, 68°41'W, 22 Jan 1994, S. Teillier 3286A (CONC).

***Seneciopygmophyllus*. CHILE. Arica-Parinacota**: cerca de laguna de Cotacotani, camino a Guane Guane, 18°10'S, 69°14'W, 9 Mar 1984, M. Arroyo 84-724 (CONC); portezuelo entre cerro Guane Guane y cerro Larancagua, 18°9'S, 69°19'W, 22 Apr 1984, M. Arroyo 84-935 (CONC); Las Cuevas, antes del Chaku, 18°11'S, 69°25'W, 20 Mar 2015, A. Moreira-Muñoz & F. Luebert 2470 (SGO); camino de Putre a Portezuelo de Chapiquiña, 18°20'S, 69°30'W, 28 Mar 1961, M. Ricardi, C. Marticorena & O. Matthei 277 (CONC); **Tarapacá**: Colchane, géiser Puchultiza, 100 m antes del géiser, 19°24'S, 68°57'W, 16 Jun 2018, A. Moreira-Muñoz 2876 (SGO). **PERU. Moquegua**: minera Quellaveco, 17°6'S, 70°36'W, 8 Apr 1999, ESCO 7238 (US); area between the carretera-binacional and the interoceanica sur, on unpaved road that connects the two main roads and borders a large bofedal, 16°51'S, 70°32'W, 12 Mar 2014, V.A. Funk, M. Diazgranados & E. Cochachin 13153 (US, USM); Mariscal Nieto, Carumas, Ancolacaya, 16°38'S, 70°19'W, Mar–Apr 2018, V. Morales 140 (USM); 5 km East of lago Suche, 16°55'S, 70°19'W, 19 Jan 1952, O.P. Pearson 5 (CONC, UC).

### New synonyms

#### 1. Senecio casapaltensis

Taxon classificationPlantaeAsteralesAsteraceae

Ball, J. Linn. Soc., Bot. 22: 47. 1885.

7947D793-8A34-522F-8332-0ACF14D3BF28

Senecio sanmarcosensis H. Beltrán, Arnaldoa 15: 211. 2009. Type: Peru. Ancash: Huari, San Marcos, Ccolla Chica, 09°40'28"S, 77°03'10"W, 5600 m, 4 May 2008, H. Beltrán 6476 (holotype: USM [USM-00277272]; isotypes: CUZ n.v., HUT n.v.), syn. nov.
Senecio repens var. taraxacifolius A. Gray [“taraxicifolius”], nom. nud. in sched. (Turland et al. 2018, ICN Art. 38.1) (US [US00829056]).

##### Type.

Peru. Lima: supra Casapalta, 4265–4360 m, 22 Apr 1882, J. Ball s.n. (lectotype, designated here: K [K000497782]; isolectotype: E [E00417028]).

##### Discussion.

*Seneciocasapaltensis* Ball was described from central Lima near the border between Lima-Junín departments, whereas the type material of *S.sanmarcosensis* H. Beltrán comes from southeastern Ancash Department. After studying several specimens from both regions, we can conclude that the differences concerning the shape and size of the leaves are not significant. The populations from Ancash tend to have a denser indumentum composed of capitate trichomes, whereas those specimens from Lima are glabrescent or the indumentum is rather deciduous and composed of shorter glandular trichomes. Nonetheless, the existence of intermediate specimens makes their recognition as distinct species inadvisable and, therefore, *S.sanmarcosensis* is here synonymized with *S.casapaltensis*.

Among the located original material of *S.casapaltensis*, the specimen at K is designated as the lectotype due to it being more complete than the duplicate at E.

#### 2. Senecio expansus

Taxon classificationPlantaeAsteralesAsteraceae

Wedd., Chlor. Andina 1: 107. 1856.

0AE8231A-ED59-5FC3-826B-7FCEA7392F17

Senecio macrorrhizus Wedd., Chlor. Andina 1: 108. 1856. Type: Peru. Cusco: dept. de Cuzco, Oct 1839–Feb 1840, C. Gay 1870 (lectotype, designated here: P [P01816797]), syn. nov.

##### Type.

Bolivia. Potosí: montagnes des lagunas de Potosí, [without date], A. d’Orbigny 1418 (lectotype, designated by Cabrera (1966) as “typus”, pg. 21: P [P01816805]; isolectotypes: BR [BR00000552801], G [G00356020], K [K000497783]).

##### Discussion.

*Seneciomacrorrhizus* Wedd. was described from Cusco (Peru) and distinguished from *S.expansus* Wedd. mainly by having a thicker, longer, and more sinuous rhizome, larger capitulum, and rosettes less spread out (Weddell 1856). The mentioned differences fall within the variability encompassed by *S.expansus*, and here we synonymize them.

Gay’s specimen P01816797 perfectly matches the protologue information, and therefore, it is designated as the lectotype of the name *S.macrorrhizus*.

### Key to the discoid caespitose *Senecio* species from Bolivia and Peru

The dwarf shrubs developing erect stems are excluded (e.g., *S.apolobambensis* Cabrera, *S.puchei* Phil., *S.trifurcifolius* Hieron.). *Senecioaquilaris* Cabrera was cited for Bolivia (Beck and Ibáñez 2014) and Peru (Gonzáles et al. 2016); it is not included in the key because its identification is doubtful and further studies are required. The rosettiform species *S.expansus* and *S.hyoseridis* (Benth.) L. Salomón & S.E. Freire were placed in S.ser.Culcitium (Bonpland) Cabrera (Freire et al. 2014; Salomón et al. 2018) but they are included in the key because they fit well within the discoid caespitose species group. The color of the anthers, style branches, and corollas has a relevant taxonomic value within the group and it is readily noticeable on living plants. However, on dried specimens a careful study is required in order to avoid misidentifications.

**Table:** 

1	Plants in rosette form	**2**
–	Plants developing prostrate or decumbent stems	**6**
2	Leaves pinnatilobate to lyrate-pinnatisect	***S.casapaltensis***
–	Leaves subentire to pinnatipartite	**3**
3	Capitula sessile, solitary or several; leaf lamina longer than or similar to the pseudopetiole	**4**
–	Capitulum shortly pedunculate, solitary; leaf lamina clearly shorter than the pseudopetiole	**5**
4	Leaves densely white tomentose on both faces, concolorous	***S.expansus***
–	Leaves only densely white tomentose beneath, discolorous	***S.hyoseridis* s.l.** (further research needed)
5	Leaves ovate-deltate, crenate-dentate, puberulous on both faces	***S.genisianus***
–	Leaves elliptic-suborbicular, subentire, with scattered long hispid trichomes above and nearly glabrous beneath	***S.sagasteguii***
6	Anthers and style branches yellowish; corolla yellowish	**7**
–	Anthers and style branches blackish; corolla whitish	**16**
7	Leaves and involucre covered by whitish lanate indumentum	**8**
–	Leaves and involucre glabrous or covered by arachnoid or pilose indumentum	**9**
8	Leaves arranged along true stems; leaves oblanceolate to oblong	***S.carhuanishoensis***
–	Leaves arranged in rosettiform clusters arising directly from rhizome-like stems; leaves obovate-spatulate	***S.evacoides***
9	Involucre 4–5 mm long; involucral bracts 8(–9)	***S.humillimus***
–	Involucre 6–12 mm long; involucral bracts (9–)12–15(–20)	..**10**
10	Achenes with indumentum	**11**
–	Achenes glabrous	**13**
11	Leaves glabrous, entire or subentire	***S.woodii***
–	Leaves covered with trichomes, dentate	**12**
12	Involucral bracts 9–12; leaves sparsely covered with trichomes	***S.moqueguensis***
–	Involucral bracts 15–20; leaves densely covered with trichomes	***S.ticsanicus*** (further research needed)
13	Leaves dentate or lobate, rarely only shallowly crenate	**14**
–	Leaves entire	**15**
14	Leaves dentate, rarely only shallowly crenate	***S.menesesiae***
–	Leaves pinnatilobate	***S.pinnatilobatus***
15	Leaves (15–)20–50 mm long, arranged along the stems	***S.algens***
–	Leaves 5–10 mm long, arranged in rosettiform clusters	***S.gamolepis***
16	Achenes papillose, with visible ribs; leaves linear	***S.scorzonerifolius***
–	Achenes silky-pubescent, usually with invisible ribs; leaves linear, linear-oblong or spatulate	**17**
17	Leaf lamina glabrous	**18**
–	Leaf lamina with indumentum	**19**
18	Supplementary bracts 4.1–5.7 mm long, a third to a half as long as the involucral bracts, glabrous; leaves linear-oblong, flat, acute at the apex	***S.madidiensis***
–	Supplementary bracts 4.2–7.5 mm long, almost as long as the involucral bracts, usually pilose; leaves linear-oblong to spatulate, usually conduplicate downwards, rather obtuse at the apex	***S.melanandrus***
19	Lamina ovate to suborbicular, differentiated from the pseudopetiole	***S.pygmophyllus***
–	Lamina linear, linear-oblong or narrowly spatulate, progressively narrowed at the base	**20**
20	Leaves dentate, pinnatipartite or distantly pinnatisect (rarely entire), arachnoid, usually with a callus-like tip	***S.digitatus***
–	Leaves entire, crenate or dentate, pilose, unadorned at the apex	***S.melanandrus***

## Supplementary Material

XML Treatment for Senecio melanandrus

XML Treatment for Senecio pygmophyllus

XML Treatment for Senecio casapaltensis

XML Treatment for Senecio expansus
